# Corrigendum: Gradient-free training of recurrent neural networks using random perturbations

**DOI:** 10.3389/fnins.2024.1511916

**Published:** 2024-11-05

**Authors:** Jesús García Fernández, Sander Keemink, Marcel van Gerven

**Affiliations:** Department of Machine Learning and Neural Computing, Donders Institute for Brain, Cognition and Behaviour, Radboud University, Nijmegen, Netherlands

**Keywords:** recurrent neural network, artificial neural network, gradient approximation, BPTT, node perturbation learning

In the published article, there was an error in Figures 5B, C as published.

The training and test sequences for this task were identical due to an issue with seed initialization in the dataset generation function. This occurred due to a mistake merging old and new code, which caused an unintentionally re-initialized seed every time a sequence was generated. Thankfully, the impact of this mistake is very small. The updated results show almost no change in overall performance. In fact, the slightly different results for this task now better align with those obtained in the other tasks (the Mackey-Glass time series and the real-world weather prediction task), further strengthening our claims about the robustness and generalization of our proposed approach (ANP).

The corrected Figures 5B, C and its caption appear below:

**Figure 5:**
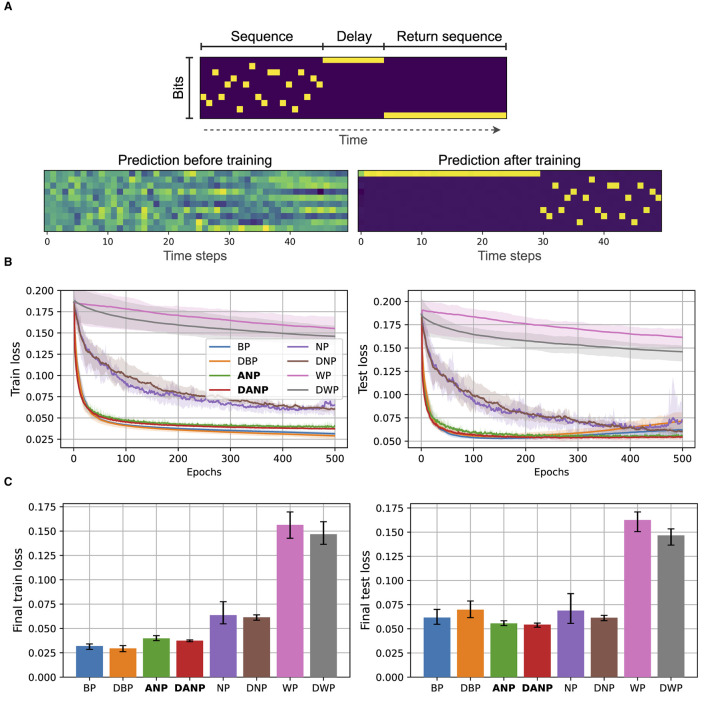
Copying memory data and results. **(A)** At the top, we depict an example of an input with annotations. The sequence length is 20 and the delay period is 10. At the bottom, we show the predictions of a BP-trained model before and after training. **(B)** Performance during training over the train and test set for the different methods. **(C)** Final performance for the different methods, computed as the mean performance over the last 50 epochs.

The authors apologize for this error and state that this does not change the scientific conclusions of the article in any way. The original article has been updated.

