# Supplementary material for: Gradient-free training of recurrent neural networks using random perturbations
Source: Front Neurosci. 2024 Jul 10;18:1439155. doi: 10.3389/fnins.2024.1439155 (PMC11267880; doi:10.3389/fnins.2024.1439155)
Supplement: Supplementary file 1 [file Presentation_1.pdf]

Supplementary Material

1 COMPARISON OF NODE PERTURBATION METHODS

We here compare the standard implementation of node perturbation with our implementation.

To this end, we compare the performance of both approaches by solving the copying memory task using the same setup as in our main experiments. The results of this experiment are shown in Figure S1. The experimental results indicate that our implementation significantly outperforms the standard node perturbation approach.

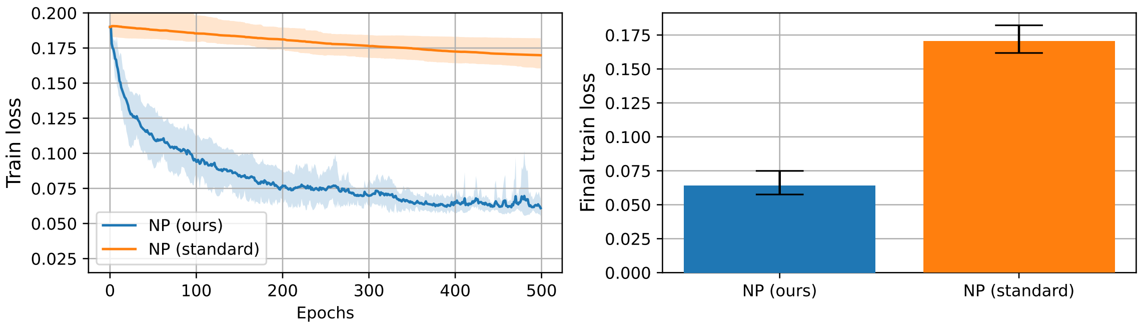

Figure S1. Copying memory task results for different implementations of NP. On the left, the performance during training is depicted. On the right, the final performance is shown, calculated as the mean performance over the last 50 epochs.

2 HYPERPARAMETER SETTINGS

In all experiments, certain hyperparameters remain fixed while others are fine-tuned to optimize the performance of each learning algorithm on specific datasets. This approach ensures a fair comparison between different algorithms. The number of epochs, hidden units, batch size, and Gaussian noise variance were kept consistent across learning algorithms for the various datasets, as detailed in Table S1. The value selected for the Gaussian noise variance is the optimal value within the range $[10^{-1}, 10^{-4}]$.

Table S1. Hyperparameters and their values used in the different experiments. Asterisks indicate that the value differs for each learning algorithm.

2***Hyperparameter**	2***Description**	**Value per experiment**		
		**Mackey-Glass**	**Copying**	**Weather**
σ^2	Gaussian noise variance	10^{-2}	10^{-2}	10^{-2}
η	Learning rate for forward and recurrent weights	*	*	*
ϵ	Learning rate for decorrelation weights	*	*	*
N	Number of hidden units	1000	500	1000
batch	Batch size	10	1	10
epochs	Number of training epochs	500	500	500

In contrast, the learning rates η and ϵ varied for each algorithm depending on the dataset, as detailed in Table S2. The values for the learning rates are the optimal values within the range $[10^{-1}, ..., 10^{-10}]$. Learning rates exceeding the depicted values yielded unstable runs, characterized by weights becoming undefined due to exploding values, or optimization getting stuck in local minima, resulting in inferior final performance. Conversely, smaller values resulted in slower learning, leading to inferior final performance. The backpropagation algorithm was employed in combination with the Adam optimizer using the default parameters.

Table S2. Learning rates η and ϵ for the different learning algorithms across datasets. Dashes indicate that the parameter is not used.

		NP	DNP	WP	DWP	ANP	DANP	BP	DBP
2***Mackey-Glass**	η	5e−4	5e−4	5e−4	5e−4	3e−6	3e−5	5e−7	5e−7
	ϵ	-	5e−9	-	5e−9	-	5e−9	-	5e−7
2***Copying**	η	5e−2	5e−2	1e−3	1e−3	4e−5	4e−5	1e−4	1e−4
	ϵ	-	1e−4	-	1e−4	-	1e−4	-	1e−4
2***Weather**	η	1e−2	1e−2	1e−3	1e−3	3e−5	1.5e−4	5e−7	1e−6
	ϵ	-	1e−7	-	1e−7	-	5e−7	-	1e−7

3 COMPUTATIONAL AND MEMORY COSTS ANALYSIS

Here, we profile the computational and memory costs of various learning algorithms considered in this paper. The analysis is conducted using the copying memory task, as it is the most studied and standard dataset in the literature. We measure the GPU operations time (in milliseconds) and allocated GPU memory (in bytes) for each algorithm on a single sample, presented one time, utilizing the PyTorch Profiler[1]. To account for potential hardware and system variability, such as GPU load, background processes, and memory management, each algorithm is analyzed five times, and the results are averaged. The GPU used for this analysis is the Nvidia Tesla T4[2]. The results of this analysis are presented in Figure S2.

Our empirical findings align with what is well-established in the literature: perturbation-based learning approaches are significantly less demanding in terms of computational and memory requirements compared to the standard gradient-based method, backpropagation. The GPU operations time is substantially lower for all perturbation-based methods considered in this study, with node perturbation being the most efficient, followed closely by weight perturbation and activity-based node perturbation. The allocated GPU memory remains comparable across perturbation-based methods, while it increases exponentially in backpropagation, particularly when incorporating the decorrelation mechanism. Unrolling the network over time with decorrelation steps results in substantial memory overhead, as all intermediate steps of all variables must be stored for each time step, unlike in perturbation-based methods.

4 COMPARISON TO ANOTHER GRADIENT-FREE METHOD

To further extend the comparison of our approach with other gradient-free methods for training RNNs, we selected random feedback local online (RFLO). RFLO shares striking similarities with our approach

[1] `https://pytorch.org/docs/stable/profiler.html.`

[2] `https://images.nvidia.com/aem-dam/en-zz/Solutions/design-visualization/technologies/turing-architecture/NVIDIA-Turing-Architecture-Whitepaper.pdf.`

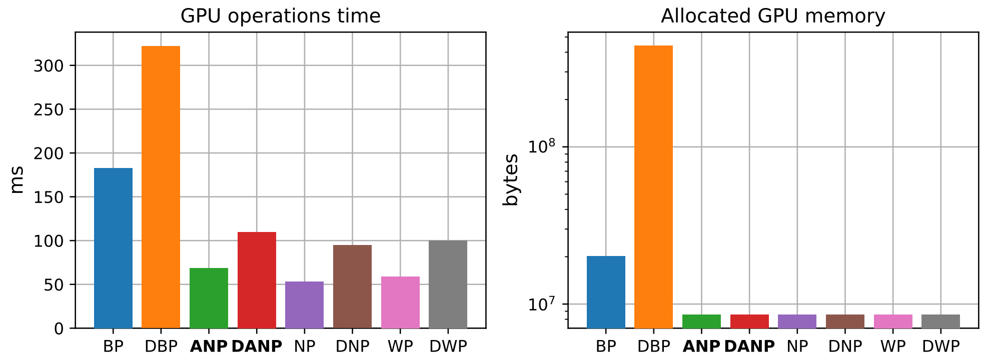

Figure S2. Computational and memory costs. Each data point represents the average of five executions. The analysis is conducted on a single sample from the copying memory dataset, presented only once. The left plot uses a normal scale, while the right plot employs a logarithmic scale for better visualization.

and demonstrates highly efficient learning coupled with computational efficiency. Moreover, it employs eligibility traces and local updates in an online setup.

The setup for the RNN model used for the RFLO is the same as the one we employed, with the only difference being that the state of the hidden neurons evolves using a time constant τ. This, while allowing for a more powerful temporal integration, adds computational complexity and leads to slower neural responses. The hidden units are defined as

$$x_t = \left(\frac{1}{\tau}\right) f(Au_t + Rx_{t-1}) + \left(1 - \frac{1}{\tau}\right) x_{t-1}. \tag{S1}$$

The weight updates are defined as

$$\Delta A = \varepsilon_t x_t, \;, \quad \Delta B = [B\varepsilon_t]\, p_t, \;, \quad \Delta R = [B\varepsilon_t]\, q_t, \tag{S2}$$

where B denotes fixed random feedback weights, ε_t denotes a local error defined as $y_t - y_t^*$, and p_t and q_t are eligibility traces capturing previous neural states defined as

$$p_t = \left(\frac{1}{\tau}\right) f'(a_t) x_{t-1} + \left(1 - \frac{1}{\tau}\right) p_{t-1} \tag{S3}$$

$$q_t = \left(\frac{1}{\tau}\right) f'(a_t) u_{t-1} + \left(1 - \frac{1}{\tau}\right) q_{t-1} \tag{S4}$$

In our experiments, the parameter τ is set to 10 as in the original work, and the learning rate is empirically selected to be the optimal value in the task within the range $[10^{-1}, \ldots, 10^{-10}]$, with 10^{-5} being the optimal value.

For simplicity, we focused on the most effective perturbation-based approaches, ANP and DANP, and the gradient-based baselines, BP and DBP. The learning efficiency comparison was conducted on the 48-hour weather prediction task, which is the most challenging task in our study. The results of this comparison are presented in Figure S3.

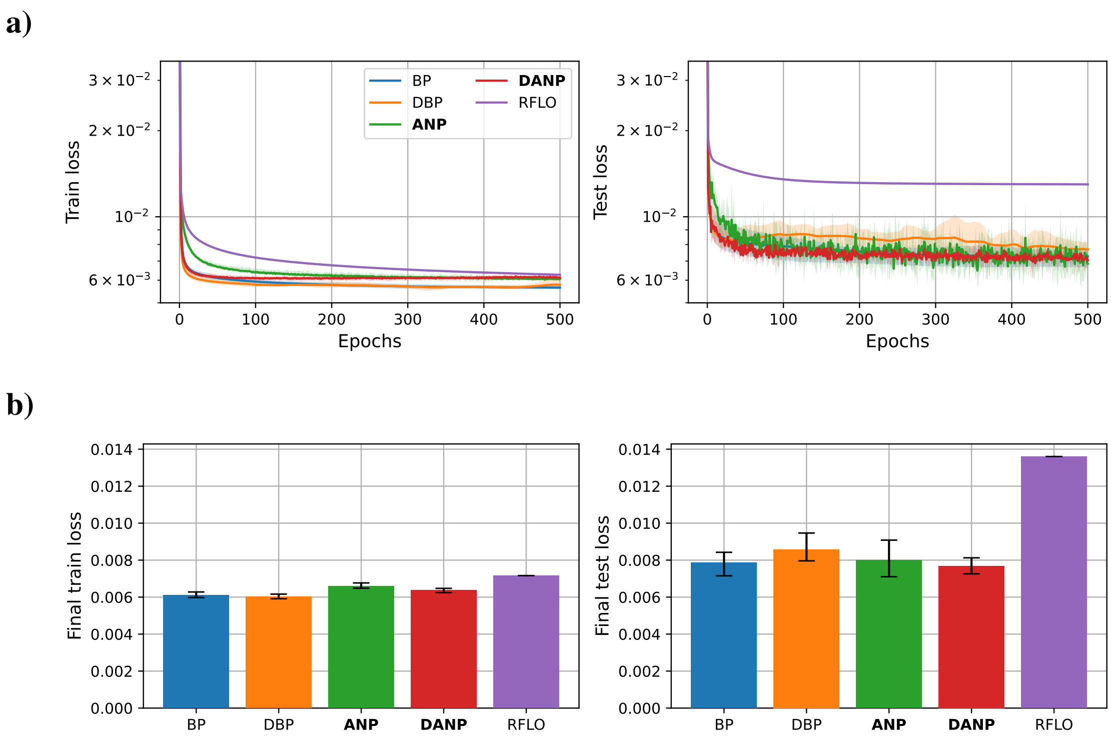

Figure S3. 48-hour ahead weather prediction results including RFLO. a) Performance during training over the train and test set for the different methods, represented in a logarithmic scale. **b)** Performance for the different methods, computed as the mean performance over the last 50 epochs.

We observe that RFLO learns the task efficiently but lags slightly behind other methods in terms of convergence speed. While its final training performance is comparable to the other approaches, its generalization capability (in terms of the test loss) is much lower.

The computational cost analysis is performed following the procedure described in Section 3 of this document. The results of this analysis are shown in Figure S4.

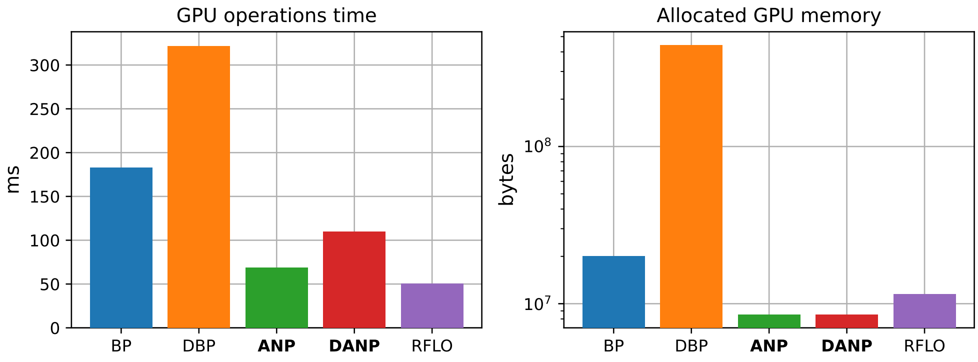

Figure S4. Computational and memory costs. Each data point represents the average of five executions. The analysis is conducted on a single sample from the copying memory dataset, presented only once. The left plot uses a normal scale, while the right plot employs a logarithmic scale for better visualization.

RFLO is slightly more efficient in terms of operation time but slightly less efficient in terms of memory usage.

5 ADDITIONAL WEATHER PREDICTION EXPERIMENTS

These experiments mirror the primary weather prediction experiments. However, in this case, the networks are tasked with predicting the target feature 1-hour and 24-hours ahead. See Figures S5 and S6, respectively.

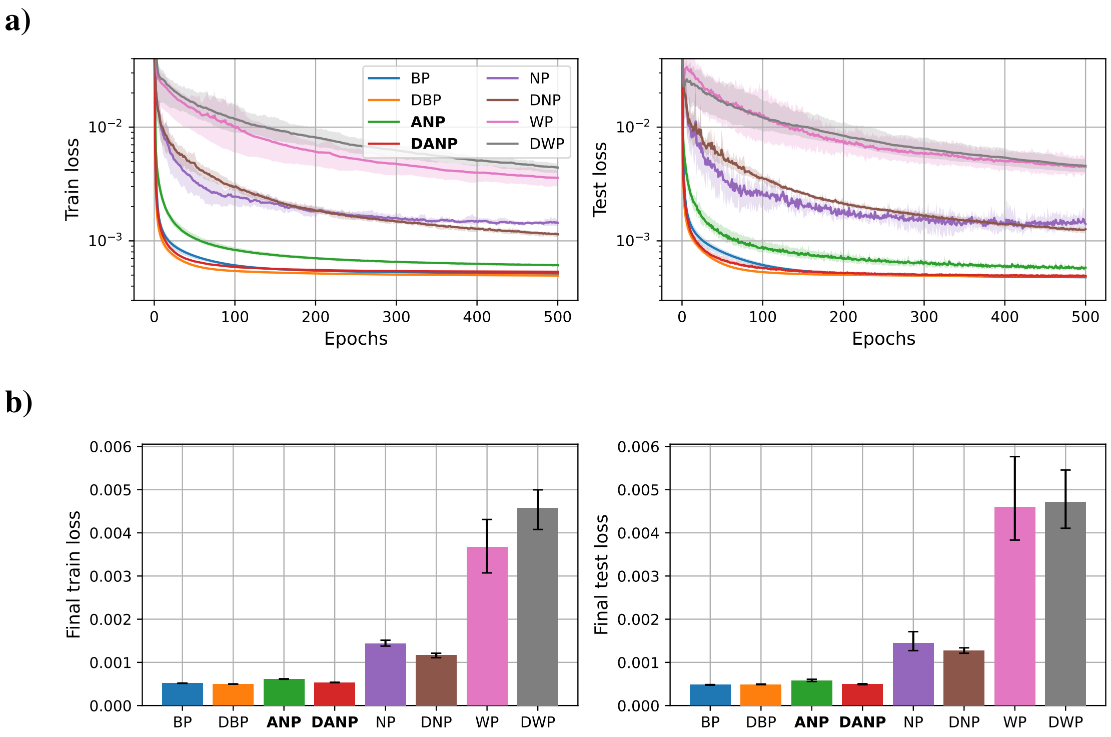

Figure S5. 1-hour ahead weather prediction results. a) Performance during training over the train and test set for the different methods, represented in a logarithmic scale. **b)** Performance for the different methods, computed as the mean performance over the last 50 epochs.

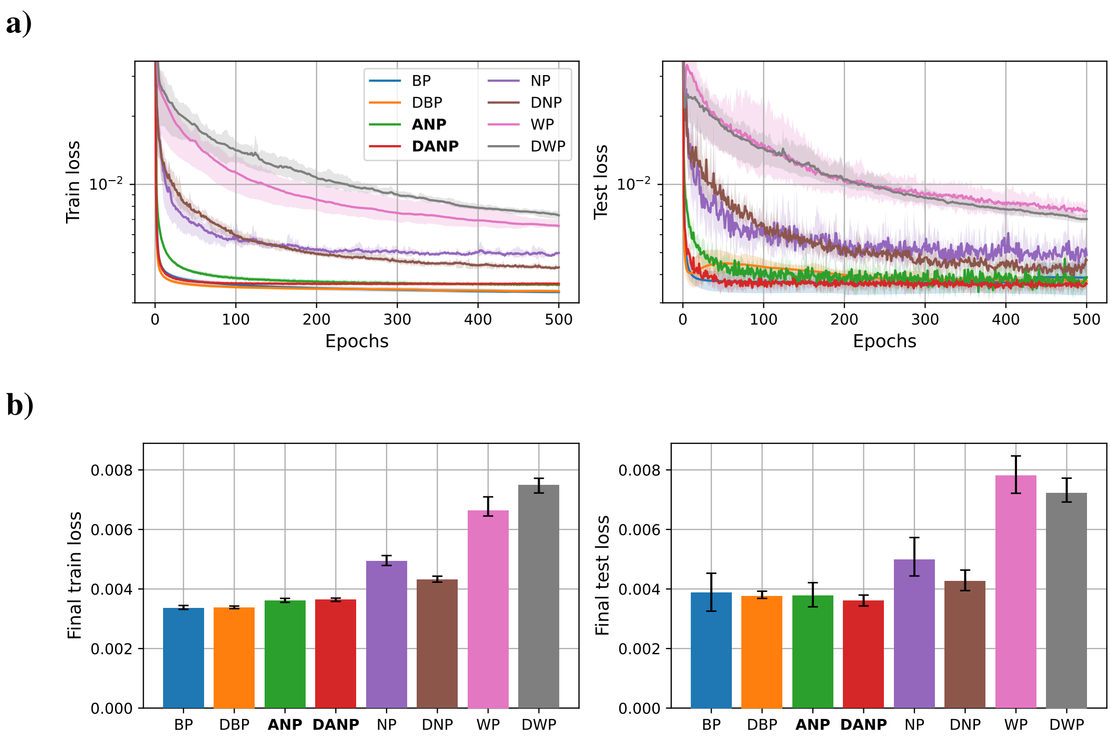

Figure S6. 24-hours ahead weather prediction results. a) Performance during training over the train and test set for the different methods, represented in a logarithmic scale. **b)** Performance for the different methods, computed as the mean performance over the last 50 epochs.
